# Spatial cognition in minimally invasive surgery: a systematic review

**DOI:** 10.1186/s12893-018-0416-1

**Published:** 2018-11-07

**Authors:** Tina Vajsbaher, Holger Schultheis, Nader K Francis

**Affiliations:** 1 Bremen Spatial Cognition Center & Department of Human and Health Sciences, University of Bremen, Enrique-Schmidt-Str.5, 28359 Bremen, Germany; 2 Department of Human and Health Sciences, University of Bremen, Bremen, Germany; 3 Department of General Surgery, Yeovil District Hospital NHS Foundation Trust, Yeovil, UK; 4 Faculty of Health and Life Sciences, University of Liverpool, Brownlow Hill, Liverpool, L69 7ZX UK

**Keywords:** Spatial cognition, Minimally invasive surgery, Surgical education, Medical cognition, Systematic review

## Abstract

**Background:**

Spatial cognition is known to play an important role in minimally invasive surgery (MIS), as it was found to enable faster surgical skill acquisition, reduce surgical time and errors made and significantly improve surgical performance. No prior research attempted to summarize the available literature, to indicate the level of importance of the individual spatial abilities and how they impact surgical performance and skill acquisition in MIS.

**Methods:**

Psychological and medical databases were systematically searched to identify studies directly exploring spatial cognition in MIS learning and performance outcomes. Articles written in the English language articles, published between 2006 and 2016, investigating any and all aspect of spatial cognition in direct relation to influence over performance or learning of MIS, were deemed eligible.

**Results:**

A total of 26 studies satisfied this criterion and were included in the review. The studies were very heterogeneous and the vast majority of the participants were novice trainees but with variable degree of skills. There were no clinical studies as almost all studies were conducted on either box trainers or virtual reality simulators. Mental rotation ability was found to have a clear impact on operative performance and mental practice was identified as an effective tool to enhance performance, pre-operatively. Ergonomic set-up of the MIS equipment has a marked influence on MIS performance and learning outcomes.

**Conclusions:**

Spatial cognition was found to play an important role in MIS, with mental rotation showing a specific significance. Future research is required to further confirm and quantify these findings in the clinical settings.

## Background

The notion that spatial cognition is a salient factor for predicting and influencing surgeons’ skill acquisition and performance in minimally invasive surgery (MIS) has already been well documented [1]. Spatial cognition however is not a unitary function but consists of a set(s) of multimodal, independent and interchangeable processes. One of such spatial cognitive abilities is Visuo-spatial ability (VSA). VSA refers to a set of abilities that allow an individual to ‘form internal mental representations of visual patterns, and use such representations to solve spatial and complex problems’ [2]. In practical terms, each of these individual and independent processes allow one to retain, retrieve and transform visual and spatial information, as according to their spatial locus. In the context of MIS, these processes govern the surgeon’s capability to, for example, a) pre-operatively position the patient according to the procedure and entry point, b) insert the trocars safely and efficiently, c) judge the spatial relation between the tip of the instruments and the adjacent organ or structure, d) apply appropriate force to the instrument, and finally, e) to successfully perform technically demanding tasks such as suturing etc.

Thus, although the impact and influence of VSA on MIS learning and performance has already been well acknowledged [3], the existing literature appears to have merely focused on investigating the role of VSA as a unitary concept. Thus consequently, despite the existence of many individual studies, it is currently difficult to pinpoint which aspect(s) of spatial cognition are most important for MIS leaning and performance, respectively. We argue that when attempting to draw conclusion about how cognitive processes impact performance, it would be advisable to start distinguishing spatial abilities from each other, as it is unwarranted to assume that they all carry the same weight of impact. Psychological literature has shown that cognitive abilities are highly mouldable by experience, presenting a convincing case that separating spatial abilities from each other could provide us with a fresh new insight into which spatial abilities form a proficient MIS surgeon. Although the findings have been largely inconsistent, most researchers seem to agree that spatial visualization (understanding three dimensional (3D) objects through two dimensional (2D) representations by creating mental representations of these objects), spatial orientation (understanding one’s own spatial relation to and with objects in space, whilst imagine what an object looks like from different perspectives) and mental rotation (mentally manipulating and rotating objects) may play a prominent role. What remains to be determined is to what extent and how these specific abilities influence learning and performance in MIS. Consequently, while the influential nature of spatial cognition in MIS is generally accepted, the knowledge regarding which of the more specific abilities are responsible for the observed influence, and exactly which aspect of performance and learning they appear to influence, remains unclear. Various studies have investigated the role of the visuospatial abilities with MIS but no prior research has attempted to summarize the evidence to identify the impact of the individual spatial abilities on performance and skills acquisition in MIS. What seems to be missing is a concise and detailed systematic analysis of the literature assessing spatial cognitive ability in MIS, in an aim to identify which specific set of abilities are important in MIS.

In this contribution, we report a systematic review of the current evidence base in aim to assess which specific spatial cognitive abilities are important in both surgical education and performance, whilst also assessing the impact that MIS related technology have on cognition. Identifying exactly which individual spatial abilities impact performance and acquisition of MIS technical skills is thus deemed fundamental for many reasons. First, the identification of specific spatial cognitive processes will allow us to devise a training program that would enable a successful outcome for novices of all cognitive levels. Such knowledge could thus have a considerable impact on the way we approach the design and implementation of the MIS curriculum. Second, the newly gained insight would also allow us to better understand the psychological attributes and profiles that underlie MIS skill learning and performance. Third, the technology continues to change and shape the surgical practice we know today. Identification of specific cognitive processes would also allow us to better understand, and even predict, which specific spatial abilities must be accounted for and trained, to allow introducing new medical technologies (surgical simulators or surgical robots) into the operative environments with minimal disruption. Fourth, a deeper understanding of the nature of the spatial abilities involved in MIS would facilitate simulator-based medical education.

## Methods

### Search strategy

We conducted the systematic review in accordance with PRISMA (Preferred Reporting Items for Systematic Reviews and Meta-Analyses) guidelines for systematic reviews [4]. We searched (October–November 2016) a variety of medical, psychological, and overall scientific databases; MEDLINE PubMed, PsychINFO, ScienceDirect, Elsevier and Web of Science. Our first search focused on spatial cognition generally using the Boolean combination of (‘spatial cognition’) AND (‘minimally invasive surgery’) and (‘spatial cognitive abilities’) AND (‘minimally invasive surgery’). Then, specific spatial abilities in combination with MIS were searched for: *Visuo-spatial abilities AND visual-spatial ability, Spatial Orientation, Mental Imagery, Perceptual-motor, Mental Rotation, Depth Perception, Spatial Perception and Spatial Memory,* e.g. ‘Visuo-spatial ability’ AND ‘minimally invasive surgery’ etc. To search for the grey literature, such as dissertation theses, Google Scholar was searched using the exact same set of search terms and combinations as stated above. Last literature search was conducted November 28th,2016, using the Web of Science database.

### Eligibility criteria

Studies were deemed eligible if they were written English and were published between 2006 and 2016. The 10-year timeframe was chosen, as we were interested in investigating developments in the literature, after Keehner et al. [5] published the noteworthy findings that spatial cognitive ability do in fact influence MIS performance exclusively. Only articles that did not directly investigated spatial cognition in MIS, or approached the topic from a purely technical perspective (engineering, for example), were excluded. To avoid publication bias and reduce positive result bias, all types of grey literature such as conference studies and theses/dissertation studies, which fitted the eligibility criterion, were included in the review. Due to the heterogeneous nature of the reviewed studies, a meta-analysis was not deemed appropriate.

### Data extraction

The data were extracted by the first author and subsequently reviewed by the second author. For each article, the following information was extracted: General research information (*First author name, year of publication, authors’ affiliations*) field of study (*psychology or medicine*)*;* which specific type of MIS procedure was explored (e.g. *laparoscopic, DaVinci*); participant characteristics (*educational level or surgical level, number of participants, age of participants*); Funding information; Methodology information (*name of the simulator, simulator task, psychometric test used*); Cognitive abilities studied; Statistics used and outcome information (Table 1). Outcomes sought after included; Operative errors, learning curves, cognitive improvement, performance outcomes and development and operative (task) time.

**Table 1:** Characteristics of the included studies

Study	Surgery	Year	*n*	Participants	Age	Field of study	Cognitive skills	Psychometric test	Simulator	Statistics	Results
Hedman et al. [10] Journal of endoscopic surgery Sweden	LAP	2006	*54*	*54* Novice surgeons (medical students in surgery training) with no previous simulator experience	Women m=24.8 Male m=25.7	Medicine + Psychology	Visual Spatial abilities correlation for novice performing complex laparoscopic simulator tasks	MT* Vanderberg and Kuse (MRT-A & MRT-C) GSA* BasIQ test	Procedicus KSA (Instrument Navigation & Manipulate and Diathermy)	Pearson’s product-moment correlation & regression analysis & paired T-test and Wilcoxon	High-level visual abilities predicted performance on spatially complex tasks on the simulator. After 10 min on a simulator, those with low abilities scored the same on the second trail as those with high abilities.
Conrad et al. [19] Journal of endoscopic surgery America	LAP	2006	*10*	Surgical residents *(n* = 6) & attending surgeons (*n* = 4) with prior simulation experience.	Not disclosed	Medicine	Mental rotation and mental scanning on camera rotation angle	None used	‘Specially constructed laparoscopic box’ stationary 10-mm, 0° laparoscope, 12 cm away from task at 35° angle was used to assess threading and knot tying skills.	Linear regression analysis, correlation coefficient	Angle increase from 15° to 90° resulted in 10–30% increase in error and time and performance. Performance decreased in directional-spatial motor tasks.
DeLucia et al. [20] Human factors and ergonomics meeting America	MIS	2006	368	Psychology undergraduate students.	Not disclosed	Psychology	Depth perception and spatial navigation	None used	E1 = Box with a customized colon + borescope E2 = Computer angle simulation (Pentium III 550 MHz)	ANOVA, mixed- ANOVA + Tukey’s (HSD)	Developing a mental model of the surgical environment and the tools pre-operatively, aids spatial navigation as it provides additional depth cues.
Hassan et al. [21] Childs nervous system journal Germany	LAP	2007	*24* F = 9, M = 14	Novice surgeons (medical students) with no previous VR experience	Mean = 34	Medicine + Psychotherapy	Spatial perception and VR laparoscopic simulator	SV* Lameris Toegepaste Natuurwetenscha ppeljik Onderzoek (TNO) SP* Stumpf-Fay Cube Prespective Test	LapSim (Surgical Science) laparoscopic simulator on cutting, clip application and coordination.	Descriptive statistics + Mann-Whitney test.	Those with higher levels of spatial perception were faster, had better performance and adopted faster to non-stereo environment than those with low levels.
Haveran et al. [23] Journal of endoscopic surgery America	LAP	2007	*24*	Experienced (Surgical residence between 3-6th training year) & medical students	Not specified	Medicine + Psychology	Perceptual distortion on laparoscopic camera and monitor positions	None used	Self designed canopy endosurgical simulator (wooden box with objects inside) with alternating angles borescope.	Used Proc mixed procedure ANOVA with Tukey’s (HSD) test Kolmogoro v-Smirnov goodness of fit.	Highlighted importance of monitor and camera positioning in laparoscopy (camera at 0° and monitor at 180°) located direct opposite the surgeon Monitor position at angles of 120° or 140° significantly declined performance Even minor camera changes decreased spatial awareness.
Hedman et al. [22] Journal of endoscopic surgery Sweden	LAP	2007	*28*	Medical students attending basic surgery course with no previous simulator experience	Mean = 27	Medicine + Psychology	Visual working memory in VR	MT* Vanderberg and Kuse (MRT-A) test	The Procedius MIST-VR PC based simulator & GI Mentor II simulator (gastro endoscopy simulator)	Pearson’s product correlation	Visual-spatial ability was found to correlate with performance, although intense training on simulator outweighed those.
Klein et al. [8] Journal of Stress and Endoscopic Surgery America	LAP	2008	*54*	Undergraduates students with no previous with endoscopic/laparoscopic simulator	Mean = 22	Psychology	Perceptual-motor abilities – effect of stress on novice performing endoscopic/lapar oscopic tasks on the simulator	ST* Dundee stress state questionnaire (DSSQ) Edinburgh Handedness Inventory	Fundamentals of Laparoscopic surgery (FLS) with McGill Inanimate System for Training and Evaluation of Laparoscopic skills (MISTELS)- including pegtransfer, circle cutting, loop placement	ANOVA with Tukeys post hoc, mixed ANOVA	Perceptual-motor disruptions are caused by the reduction in depth information and transformed spatial mapping, responsible for reducing the performance on the endoscopic or laparoscopic simulator. The loss of depth information and disrupted eye-hand coordination was found to increased stress.
Hsu et al. [24] Journal of Endoscopic Surgery Canada	LAP	2008	*40*	27 novice and residence, 9 experienced – 5 fellow/staff, 4 PGY 3-3y)	Not specified	Medicine	Cognitive decisions and technical skill automatization when using cognitive distractions (mathematical algorithmic questions during simulator.	None used	FLS simulator (lighted, enclosed laparoscopic trainer box in a fixed position)	Descriptive statistics, Student’s T-test	Cognitive distraction reduced the performance of a novice, but not an experienced surgeon (Due to the technical of the task).
Keehner [3] Spatial Cognition United Kingdom	LAP	2008	40 F = 22, M = 18	Undergraduates Psychology students	Mean = 20	Psychology	Visual-spatial abilities and frame of reference	MT* Mental rotation test, The paper folding test and Card rotation tasks.	Self-made simulator to mimic laparoscopic conditions using laparoscopic two camera angles (90° and 270°)	Independen t samples T-test,, one samples ttest, correlationa l analysis	Seeing the back of your hand in a 90° o the monitor increased performance. 270° angle decreased performance
Komesu et al. [14] American Jo urnal of Obst etrics & Gyn ecology America	ENDO	2009	*68*	Surgical residents	Control group M = 29.3 Imagery group M = 28.7	Medicine	Mental imagery practice in improving preoperative cytosopic procedure (minimally invasive endoscopic procedure)	The Global Scale of Operative Performance (GSOP)	None used	Power analysis, T-test for continuous variable, Fisher exact for categorical variables, Wilcoxon rank for ordinal variables & ANOVA.	Residents who practiced mental imagery preoperatively, showed superior performance as compared to the control group. Suggested that mental imagery would show greater effect if performed prior to complicated procedures which involve complex cognitive components.
Sodergren et al. [13] The Annals of Surgery United Kingdom	LAP	2010	*21* M = 19, F = 2	Surgeons (4 attending surgeons, 3 senior residents and 14 junior residents)	Mean = 31.6	Medicine	Spatial orientation and strategies in laparoscopic cholecystectomy	None used	Used eye-tracking Tobii ET 1750,an infrared video-based binocular eye tracking system.	Non parametric Kruskal Wallis test and Mann Whitney U test Coefficient correlation of determinati on	Laparoscopic surgeons create discernable visual strategies to orientate themselves. The homogeneity of performance suggested that laparoscopic surgeons do at some point reach a plateau consistent with their innate abilities. Experie nced surgeons made spatial disorientation errors 22% of the time. Age and gender were found to be predictors of performance.
Arora et al. [15] American college of Surgeons United Kingdom	LAP	2009	*18*	Novice surgeons recruited by random sampling.	Mental practice group = mean 22 Control group = mean 22	Medicine	Mental practice and indirectly mental imagery to reduce stress when training novice surgeons on a laparoscopic VR simulator	MT* Mental Imagery Questionnaire (MIQ) ST* Imperial Stress Assessment Tool (ISAT), State Trait Anxiety Inventory (STAI)	MIST-VR simulator used to compare participants The LAP mentor VR laparoscopic surgical simulator used for the actual task	Descriptive statistics, Mann- Whitney U test, Spearman rho correlation	Performing a short mental practice training preoperatively was found to reduced stress intraoperative, both psychologically and physiologically. Mental practice was found to improve cognitive skills.
Zhang et al. [18] Proceedings of the Human Factors in Ergonomics Society America	LAP	2010	*24*	Laypeople with no prior experience in performing laparoscopic surgery or VR related simulation	Between 22 and 45	Human factors & Ergonomics	Effects of visual-motor misalignment on laparoscopic surgery performance	None used	DynaMITE simulator, consisting Stryker Endoscopy system and the Dynamic Minimally Invasive Training Environment (DynamMITE)	A 2-way ANOVA with Tukey’s HSD post hoc analysis using a Bonferroni adjustment.	Performance was best when the image was rotated at 0°. Performance was best with endoscope at −45° and worst at 180°.
DeLucia et al. [17] Journal of Experimental Psychology America	LAP	2011	*36*	Psychology undergraduate students who received course credit.	Not stated	Psychology	Effect of camera arrangement on Perceptual motor performance in MIS	None used	Self made wooden box with bullet cameras and surgical graspers	Mixed ANOVAs with Tukeys HSD	Viewing an image from the camera perspective degraded performance, compared to direct viewing.
Sodergren et al. [13] British Journal of Surgery Society United Kingdom	LAP	2011	*30* M = 21, F = 9	Medical students (Final year)	Control Group (median = 23), Intervention Group (median = 22)	Medicine	Spatial orientation in laparoscopic cholecystectomy	None used	No simulator used – used laparoscopic video Used eye-tracking Tobii ET 1750,an infrared video-based binocular eye tracking system.	Kurskal- Wallis test with Mann- Whitney	Teaching orientation strategies to novice surgeons significantly increases their performance and reduces the cognitive burden
Kolozsvari et al. [25] Journal of Endoscopic Surgery Canada	LAP	2010	*32* M = 19, F = 13	Medical and dental students with no previous surgical experience	Mean = 23	Medicine	Exploring gender differences on laparoscopic surgical skill acquisition whilst testing for visual-spatial, spatial orientation, spatial scanning & perceptual abilities	VS* The Ekstrom-French Kit of Factorreferenced cognitive test SPT* Card Rotation and Cube Comparison test. SS* Map planning test SP* Pictorial Surface Orientation (PicSOr)	Fundamentals of Laparoscopic surgery (FLS) on a peg transfer, circle cut, placement loop and tying. Controlled for handiness (*r* = 30, l = 2), interest in surgery (high = 12, moderate = 15) and video game experience	Used SPSS Nonlinear regression, ANOVA and univariate analysis factors	No gender difference was found, indicating that gender does not affect the learning curve. Interest in surgery and perceptual abilities did influence the early-simulated performance only.
Klein et al. [8] Journal of Stress and Endoscopic Surgery America	MIS	*2012*	*15* M = 13, F = 2	First year medical students	Mean = 25	Psychology	Mental workload and Stress perceived by novice surgeons in Laparoscopic and Robotic surgery	MW* Multiple Resources Questionnaire (MRQ) ST* Dundee Stress State Questionnaire (DSSQ)	Fundamentals of Laparoscopic surgery (FLS) trainer box with a DaVinci surgical system – on a peg transfer task	Descriptive statistics, Bonferroni-corrected t-test, a 2 × 7 and 2 × 11 ANOVA.	The Da Vinci system allowed for an overall better performance compared to the laparoscopic system. No difference in mental workload score was found between the two systems, although the DaVinci did reduced stress.
Luursema et al. [28] Learning and Individual Differences The Netherlands	LAP	2012	*24* F = 19, M = 5	University students in Technical Medicine (participation in this study was required as part of the course).	Aged either 21 or 22	Medicine + Psychology	Exploring visual-spatial, Spatial relations, flexibility of closure and perceptual speed abilities on duration, motion efficiency and damage on the laparoscopic simulator tasks.	Demographic questionnaire VS* Vandenberh and Kuse test SR* Cards test FC* Hidden Objects test	LapSim v.3.0.10 simulator with Immersion VLI hardware, running on PC on grasping and instrument navigation tasks. Training course lasted for 2 months (8 weekly for 30 min)	Repeated measures analysis, repeated measures ANCOVA (Mauchly’s test of sphericity was not assumed)	Visualization abilities impacted performance on damage and motion. Perceptual speed only predicted the speed factors and not complexity. Training on the simulator outweighed the innate visual abilities.
Mistry et al. [9] Journal of Surgical Education Canada	LAP	2013	*31*	First-and-second year medical students with no laparoscopic experience.	Not stated	Medicine	Visual-spatial abilities and manual dexterity (in connection to stereoscopic vs. monoscopic) effect o surgical skill acquisition in novice surgeons.	VS* Vandenberg and Kuse Mental Rotation Test Manual dexterity* Purdue Pegboard Test Fine and Gross Stereoscopic vision* Schmetterlings Test and Graded Circle Test (KAVITA)	Fundamentals of Laparoscopic surgery (FLS) with McGill Inanimate System for Training and Evaluation of Laparoscopic skills (MISTELS)- including peg-transfer, circle cutting, loop placement	Data tabulation, MANOVA, correlation coefficient	No significant difference between the stereoscopic and monoscopic vision on laparoscopic tasks was found, except in peg-transfer where monoscopic visualization was found to improve performance. External stimuli (haptic or auditory) did increased the cognitive load (mental effort)
Roach et al. [12] Anatomical Sciences Education Canada	LAP	2013	*20* M = 13, F = 7	First-and-second year medical students with no previous surgical specific and no laparoscopic experience. Uses the sample from Mistry et al., 2013	Mean = 23	Medicine	Visual-spatial abilities and laparoscopic skills in novice surgeons, comparing stereoscopic and monoscopic visualizations.	MT* Vandenberg and Kuse MRT-A test Vision* Stereo Butterfly test and Graded Criclr test	Fundamentals of Laparoscopic surgery (FLS) with McGill Inanimate System, including peg-transfer, circle cutting, loop placement	Data tabulation, ANOVA, correlation coefficient	Those with high visual-spatial (HVs) outperformed those with lower-spatial abilities (LVs) and gained technical skills more rapidly.
Louridas et al. [16] British Journal of Surgery Society Canada	LAP	2014	*20*	Senior surgical trainees (Postgraduate year 3 and 4 general surgery residents)	Not stated	Medicine	Mental practice in enhancing laparoscopic surgical performance	MP* Mental Imagery Questionnaire Revised second version (MIQ-RS) ST* State-Trait Anxiety Inventory (STAI) Non-Technical Skills for Surgeons (NOTSS)	Self-made box trainer, using a porcelain bowel model	Nonparametric tests, Wilcoxon rank sum test and Mann-Whitney U test.	Mental practice (with script and voice-over) improved mental imagery and advanced laparoscopic technical skill acquisition. Surgeons who practiced mental practice showed better response to intra-operative stress.
Groenier et al. [26] Advances in Health Sciences Education The Netherlands	LAP	2014	*53*	Undergraduate students in Technical Medicine program with no previous laparoscopic experience.	Mean = 22	Multidisciplinary (Science and Technology, Psychology and Medicine)	Exploring the relationship between spatial memory, perceptual speed and general reasoning ability in laparoscopic simulator training	VS* Vandenberg and Kuse, Paper Folding test, the surface Development test and The Rotating Shapes test. SM* Corsi Block Tapping test PS* The Number Comparison test, Identical Pictures test Global Reasoning: Raven Advance Progressive Matrices test Verbal Reasoning: Groninger Intelligence Test	LapSim v.3.0.10 Surgical Science using Immersion’s VLI hardware	Correlation coefficient, MANCOV A & Regression analysis	No relationship between cognitive aptitude, duration of training or steepness of the learning curve was found. Visual-spatial and reasoning abilities were associated with performing a task faster. Perceptual speed was to be found positively associated with efficiency of moments, whilst spatial memory and perceptual speed were associated with the amount of damage.
Utesch [6] Behavioral Sciences and Cognitive Psychology	LAP	2014	*28* M = 3, F = 25	Psychology University students with no prior laparoscopic experience	Mean = 22	Cognitive Psychology and Behavioral sciences	Exploring the relationship between visualspatial, spatial memory, reasoning ability and processing speed and the VR laparoscopic simulator	SR* Raven’s Progressive Matrices MT* Paper Folding Test, Vandenberg and Kuse SM* Corsi Block Tapping Test PS* Identica Pictures tests SR* Rotating shape test PA* PicSOr test	LapSim simulator with a LapSim 2013 software on cutting and clip applying tasks (under difficult level)	A multiple regression analysis, Linear regression	A weak relationship between all cognitive aptitudes and the initial performance and errors made was found The perceptual speed strongly correlated with the time taken to complete the task and tissue damage.
Fan et al. [7] Journal of Endoscopic Surgery The Netherlands	MIS	2014	*24* M = 12, F = 8	Undergraduate and PhD students with no previous experience with minimal invasive surgery and the Endo- PaC simulator	Mean = 25.5	Biomechanical Engineering	Investigating two effects of spatial disorientation – “control-display compatibility” and “local disorientation” in minimally invasive surgery	A performance questionnaire	Custom-developed Endo-PaC simulator was + custom designed software with a 3D-curved tunnel	One-way repeated measures analysis ANOVA with post hoc & One-way independent ANOVA with post hoc & paired t-test	A visible endoscopic camera on the monitor improved performance, workload and path length by serving as a guide regarding the direction of the instrument. This ultimately improved the spatial orientation of the surgeon.
Groenier et al. [27] Journal of Surgical Education The Netherlands	LAP	2015	*98* M = 46, F = 52	Undergraduate student in Technical Medicine with no previous laparoscopic experience. Participation was required as part of a curriculum. Combined a sample of 53 students taken from Groenier et al. 2014 study	Mean = 23	Medicine	Study the influence of both cognitive and psychomotor abilities on the training duration and learning in novice practicing laparoscopic tasks.	VS* Vandenberg and Kuse test, the Paper folding test, the Surface development test and the Rotating shapes test. SM* The Corsi block tapping test PS* the Number comparison test, Identical pictures test SR* The Raven advanced progressive matrices test, Groninger intelligence test	Immersion’s VLI hardware with LapSim simulator	Descriptive statistics Kaiser- Meyer- Olkin measure of sampling adequacy & Principle component analysis, Cox proportional hazards model	Found perceptual speed and psychomotor ability to successfully predict the rate of skill acquisition on a laparoscopic simulator. Those with higher PC abilities reached skill proficiency fater, than trainees with lower PS. No relationship between VS abilities and performance was found.
Schlickum et al. [11] International Journal of Medical Education Sweden	LAP	2016	*30* F = 12, M = 18	Medical students with no experience in VR and high motivation for surgery	Mean = 25	Clinical Sciences and Psychology	Exploring if Visual-Spatial abilities predict performance, and if surgical simulation performance and previous video gaming experience correlates with motivation to further train on a simulator.	Demographic questionnaire VS* Vandenberg and Kuse MRT-A test Situation Motivation Scale (SIMS)	Minimal Invasive Surgery Trainer Virtual Reality (MIST-VR) simulator using the manipulative diathermy medium task.	Power analysis, student t-test, MANOVA, Shapiro Wilk’s test, Regression analysis, Pearson correlation coefficient	Visual-spatial ability was found to be more important than motivation for predicting performance on the simulator Previous video game experience showed a correlation between simulator training and motivation.

Note: The findings presented in this table were precisely reproduced as originally reported by each individual study

Abbreviations: Surgery: *MIS* Minimally invasive surgery, *LAP* Laparoscopy, *ENDO* Endoscopy. Psychometric test: *VS* Visuo-spatial, *MT* Mental rotation, *GSA* General spatial cognitive, *SV* Stereoscopic vision, *SP* Spatial perception, *SPT* Spatial Orientation, *SS* Spatial Scanning, *SR* Spatial Relations, *FC* Flexibility of closure, *MW* Mental Workload, *MP* Mental Practice, *SM* Spatial Memory, *SR* Spatial Reasoning, *PA* Perceptual Ability, *PS* Perceptual Speed, *SR* Spatial Reasoning, *ST* Stress Test

### Study selection

Articles were screened from titles and abstracts by two independent reviewers (TV & HS). Studies, which were found to satisfy the criterion, were then screened for full-text in greater depth. Studies that carried a heavy engineering focus (e.g. testing how a software can track spatial misalignment) or investigated spatial cognition as a secondary factor (not directly testing or exploring spatial cognition in MIS performance and/or learning) were excluded from the review. Further articles were excluded if they had duplicates, incomplete data or were not available in full text.

## Results

**Fig. 1:**
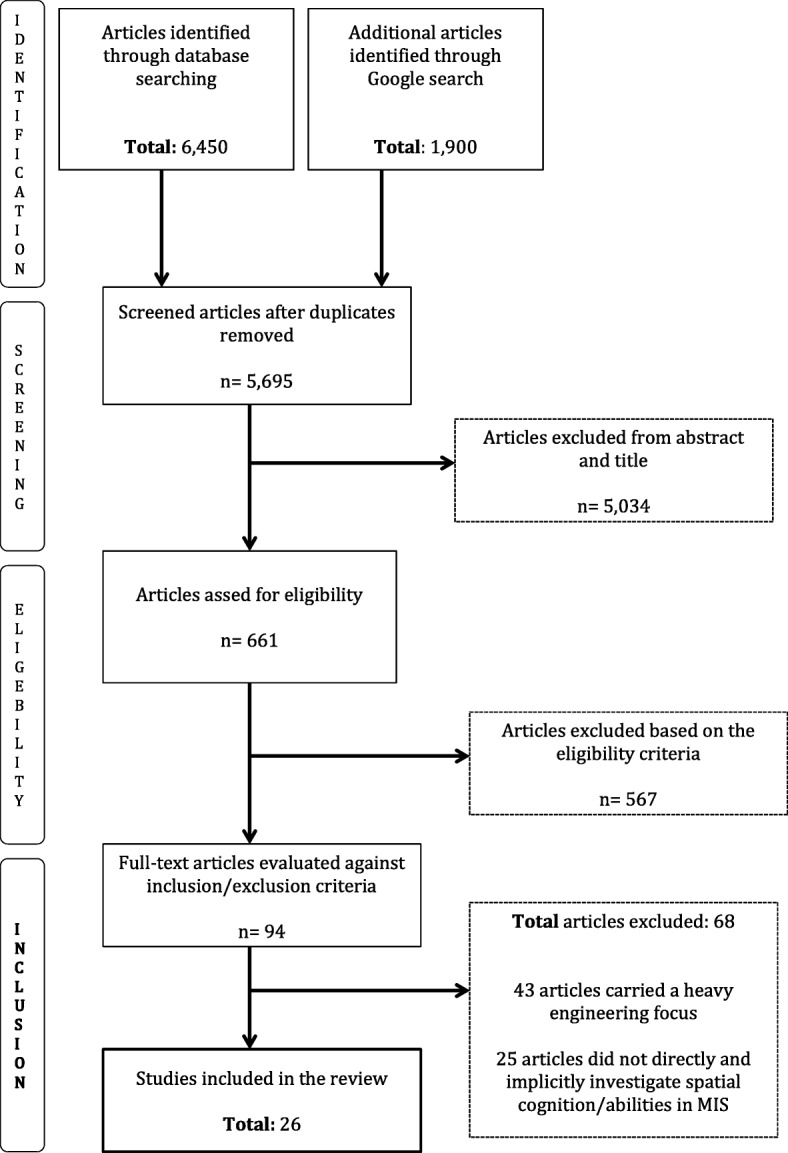
Flowchart showing study selection process

A total of 6450 articles were identified through databases, and 1900 articles through Google Scholar. After carefully screening and removing the duplicates from the databases, the remaining 5695 articles were screened based on their titles and abstracts. Through this process, 661 articles were screened against the eligibility criterion, of which 567 full-text articles were identified. Out of the 567 full-text articles, 94 articles were again evaluated against the inclusion and exclusion criteria (stated above). Sixty-eight identified articles were excluded as 43 articles carried a heavy engineering focus (e.g. describing guidance system using CT scans to improve the visual-spatial information processing intra-operatively), whilst the rest of the 25 articles were excluded as they did not explore or investigate spatial cognition, or specific spatial cognitive abilities (i.e. depth perception) in direct relationship to MIS performance and/or learning. Finally, mere 26 articles were found to meet the eligibility and inclusion criterion and were included in the review (Fig. 1). To identify any relevant grey literature on Google Scholar, the list of excluded and included articles from the databases were used to search for new and relevant articles. Through this process, five grey literature articles were identified, of which one bachelor thesis [6] was found to meet the eligibility and inclusion criterion, and was included in the review.

### Included studies

Of the 26 included studies, 22 were correlational studies exploring relationships between spatial cognition/abilities and either performance and/or learning outcomes in MIS whilst the remaining four studies were comparative studies, aiming to compare the effect of/on spatial cognition/abilities on specific technical characteristics in MIS (e.g., comparing the effects of spatial disorientation on MIS performance by manipulating the visibility of the endoscopic tip on the monitor [7–9]. Of the 26 reviewed studies, four studies focused primarily on investigated visuo-spatial ability [3, 10–12], two investigated spatial orientation [7, 13], three investigated mental imagery/practice [14–16], three investigated perceptual and visuo-motor ability [8, 17, 18], one investigated mental rotation [19], one depth perception [20], one spatial perception [21] and another visual working memory [22]. Further seven studies investigated a combination of one or more of the above-mentioned spatial abilities, or investigated ‘spatial cognition’ in its general term [23–26]. The heterogeneous nature of the reviewed articles is further illustrated in Table 1. Overall 1214 participants were included in the reviewed literature, out of which 673 were undergraduate university students (enrolled in either psychology of translational medicine course), or medical students, 353 surgical residents (largely in the first year of training) and 13 qualified surgeons (attending or consultant).

### Main findings

#### VSA as a predictor of simulator performance

All studies in this review investigating the relationship between VSA and MIS performance and learning, found VSA to have an impact on operative simulator performance. These studies administrated the mental rotation test (MRT) to assess the individuals’ VSA. Nevertheless, they were inconsistent in regards to which element was found to be most prominent, the innate VSA [3, 10–12], or the mere practice gained on the simulator [27]. Only two studies attempted to link VSA with a specific aspect of simulated MIS who also reported inconsistent findings, with one study arguing that VSA only predicts damage and motion on the simulator [28] and other that VSA only predicted the task completion time [27]. The targeted population of these studies were mainly young students, or young resident surgeons with a maximum of 2 years of experience, with an average age of 24 years. A large majority of these studies targeted medical students [9–11, 21, 22, 25, 28], or even psychological undergraduate students [3, 6, 17, 20]. Two studies [10, 12] employed a between-subject design and divided students into two groups, depending on their levels of innate spatial abilities. Prior video game experience was found to positively impact performance and improve mental rotation skills [11] whilst additional haptic and auditory cues were found to negatively impact performance [9].

#### Mental practice and mental imagery impact MIS performance

All three studies who aimed to explore the impact of mental practice on MIS performance, both in simulated and real-life environment, found mental practice and its form of mental imagery to enhance surgical performance if used pre-operatively [14–16]. One study [14] reported that residents who practice mental imagery pre-operatively performed considerably better on a real cystoscopy procedure than residents who did not, whereas the other two [15, 16] found mental practice to improve mental imagery, which in turn improved performance, technical skill acquisition and better response to crisis and stress.

#### Impact of ergonomics on VSA and MIS performance

The VSA is influenced by the technical setup which is ultimately impact surgical performance. The impact of technical setup was measured in terms of angularity, where research attempted to identify an optimal angle of both the endoscopic camera and monitors. All studies agreed that increased degree of image rotation decreased performance, although the conclusion in regards to the optimal degree of angle varied. One study found endoscopic cameras should be held at 0° angle, with no rotation [18, 23], whilst another concluded that the combination of 90° camera angle and surgeons direct hand view on the display increased performance. Monitor displays were found to increase performance if they were located directly opposite the surgeon, at a 180° angle [23], as it aligned visual view with cognitive spatial motor movement [17]. Reduction in performance was reported with camera rotations of 270° angles [3] and between 15° to 90° angles, which lead to 10 to 30% increase in error due to decreased directional spatial-motor ability [19]. Conrad et al. [19] was the only study that tested the degree of angularity on experienced surgeons and residents who already have experience with real-life laparoscopy procedure. Perceptual-motor disruptions were reported to be a result of decreased depth information in spatial mappings, reporting that the loss of depth information inhibits the eye-hand coordination leading to stress [8]. One study proposed that having a visible endoscopic camera tip going in the same direction as the hand, significantly increased performance by decreasing the cognitive load (the amount of mental effort required to perform a task) [7].

## Discussion

Spatial cognition appears to be a prominent constraint for the acquisition and performance of MIS skills, although the role of specific cognitive parameters remains debatable. Thus, what seemed to be missing was a concise and detailed systematic analysis of both medical and psychological literature assessing spatial cognitive ability in MIS. The aim was to better understand what role spatial cognition, and the specific underlying processes, plays in influencing, supporting and even predicting surgeon’s performance and skill acquisition in MIS techniques.

Our review of the existing research literature on the topic of spatial cognition in MIS indicates that VSA plays a significant role in the acquisition of MIS technical skills in a simulated environment, but mainly in inexperienced novices. The review also highlighted that novices with stronger VSA, or more specifically a stronger mental rotation (MR) ability, can acquire the required technical skills at a faster rate. Additionally, mental practice and mental imagery were also identified to play a significant role of enhancing performance of more experience surgeons when preoperative practice is carried out. This review has also identified the importance of considering both internal and external factors that may influence and interact with performance such as ergonomic set up. Surgeon’s own spatial cognitive abilities appear to be negatively impacted by ergonomic factors, such as angular discrepancies in technical setup of the monitors, which would further reduce the novice’s cognitive capacity and thus directly hinder their learning and performance.

The current literature, however yielded no substantial insight into which specific VSA processes appear to be most important, mostly due to extensive use of the MR test. These findings further highlight the greater need for a more holistic research approach, where the impacts of external and internal factors on the surgeon are more carefully considered. Furthermore, literature demonstrated that both physical and virtual surgical simulators are effective methods of acquiring MIS related technical and cognitive skills. Although research has shown technical skills to be transferable from the simulator into the OR, the same cannot be said for the cognitive skills. Considering the influential role of visuo-spatial processes, or more specifically the mental rotation in the acquisition of MIS skills, establishing the transferability from the simulators into the real life OR remains critical and to be proven.

Considering the role of MR alone does not seem to provide an explanation as to why mental practice influences intraoperative performance, or why MR diminishes in importance with practice [10]. Most importantly, by focusing on specific cognitive processes, such as MR and VSA alone, which carry high individual and environmental variabilities, we directly hinder our efforts in informing and designing MIS training curriculums or intraoperative system to suit individual training needs. This is especially critical when considering the environment in which these surgical skills ought to be acquired, as we know that external influences also carry further cognitive and behavioural consequences [29]. Thus, if wishing to use the knowledge of cognition to actually benefit the present and future generation of clinicians, we must take a holistic and broader new perspective. This can be achieved by more closely considering the functioning of a much larger cognitive network, the working memory (WM), or more specifically the visual working memory (VWM). We will provide a theoretical narrative of this new perspective, and its direct clinical implications, below. Before that, we discuss a number of shortcomings that ought to be considered when interpreting the findings of the existing studies, such as lack of expert data, inconsistent experience classifications, underrepresentation of the over 40 years age group, and unrealistic experimental simulator settings.

### Methodological limitations of the reviewed studies

Firstly, out of 1214 participants from all 26 reviewed studies, only 13 of them were experienced surgeons (attending or consultant surgeons), which either acted as a comparison group or were used as a method of judging performance. Given the extremely small sample of the expert data, it is simply impossible to infer any insight into what the performance of novices ought to be like. This poses a particular challenge for current and future studies on the topic, as we currently do not have enough empirical evidence to allow us to understand what actually makes a proficient MIS surgeon and how this informs surgical education.

Secondly, labels such as ‘novice’ and ‘experienced’ to describe individuals’ expertise levels were used heterogeneously throughout the literature. For example, Haveran et al. [23] placed medical students under ‘novice’, Arora et al. [15] places surgical trainees, who assisted in ORS but have not done the procedure alone under ‘novice’ whilst Hassan et al. [21] provided no account on the experience level of the participants, except that they all had no previous simulator experience. This is a crucial factor that must be kept in mind when making sense of the novice data, as there seem to be variations of experience levels within that category. This also calls for future research to design a classification criterion where specific factors of experience are accounted for.

Thirdly, considering that a large majority of studies investigated cognition in the context of education, it is unsurprising that the average age of all participants in this review is 24. This trend was also seen in the experienced surgeon’s group, where the oldest participant 38 years old. This is particularly astonishing, as research on spatial cognition has clearly identified age as being an important factor to predict surgical performance [27]. Medical professionals were found to have generally low self-assessment capabilities, suggesting that they have limited self-awareness capabilities in recognizing their own cognitive decline [30].

Finally, the transferability of technical motor skills from a simulator has been documented [31], but the extent to which cognitive abilities are transferred remains unanswered. Thus, crucially, the transferability of the cognitive skills from the simulator in the OR remains unknown. In most studies in this review, individuals were trained on a simulator alone without any guidance and lack of feedback. This provides a student with an unrealistic experience, as performing surgery is much more of a team effort than an individualistic mission. This is especially the case in MIS, where it is common to have up to three supporting surgeons operating at the same time. Researchers are encouraged to take advantage of physical simulators as means to study the impact on cognition if team effort is required. Ambient intelligence, for example, has the means to design a much more realistic virtual environment, through the use of sensors, to train surgical trainees in a more realistic manner.

#### A new approach to surgical education research

Current literature on spatial cognition in MIS surgical training/performance shows VSA to have a clear impact on MIS learning and performance. Nonetheless, due to several identified methodological limitation in the existing literature, it is currently difficult to infer how to best utilize such knowledge to support MIS skill learning and promote the efficient intraoperative performance. Thus perhaps, it may be worthwhile to take a more holistic approach and consider the functioning of a much larger cognitive system, which is directly responsible for mediating all of the identified cognitive processes. This system is *working memory*, with a specific focus on *visual working memory*. We argue that by considering a more central role of WM as a whole, we could make better sense of the fragmentary findings in the literature. In this section, we will provide an argument as to how theoretical knowledge of the WM could help us answer these persisting questions, and advance our efforts for promoting efficient intraoperative learning and performance.

First and foremost, the concept of WM is used to describe an active ‘mental workspace’, which allows us to temporarily process all incoming stimuli encountered in the environment, helps us to maintain focus on what matters, helps to block out unnecessary information and finally, delegates the activation of specific cognitive processes required for the execution of a specific task [32]. The component of WM responsible for processing and manipulating visuospatial stimuli is referred to as VWM. As one example, just think of a novice surgeon who is learning the laparoscopic technique. In order for the surgeon to perform the technique safely and effectively, a step-by-step intraoperative procedure (e.g. positioning the patient, correct insertion of the trocars etc.) and associated knowledge, must be retained and maintained in the WM. When navigating to the targeted lesion inside the body, the surgeon must selectively and attentively attend to the task-specific visual and spatial information of the structures and tissues, whilst mentally manipulating the 2D image seen into the actual 3D representation of the human anatomy. The surgeon must then actively retrieve important operative knowledge (both patient and procedure specific, for example) from his short and long-term memory, whilst maintaining all of the currently relevant information. Finally, the surgeon must then also continue to track and monitor the coordination of his own instruments in hand in relation to the instruments of his assistants and the camera view, in an aim to perform a specific operative task. All of these individual activities and processes require simultaneous processing in the already limited ‘workspace’ within the WM, whilst appropriately mediating and recruiting relevant cognitive processes to allow for efficient task-specific behavior [33]. The impact of WM on operative performance has mostly been measured through eye-gaze analysis. Most of these differences lay in the gaze fixation, with expert surgeons fixating and maintaining a direct gaze of the operative field and novices showing a repeated gaze switch between the target and the movement of the instruments [29]. Through this continued gaze switch between the monitor and the instruments, novices take in significantly more perceptual information within the environment, placing a higher demand on their attentional resources. This inevitably leads to further reduction of their overall WM capacity, leading to cognitive overload [29, 32]. The concept of cognitive load refers to reduced WM capacity during learning, where through the inappropriate allocation of attention of various internal (thought process) and external information (environment), the novice attempts to process and retain multiple incoming cues and stimuli’s. Such notion can be described through the following formula: The more difficult or unfamiliar the task is, the more attention and cognitive resources must be attended to thus the greater the demand on the WM is [29].

#### Clinical implication

In this article, we propose a new approach to study the role of spatial cognition in MIS learning and performance. We argue that by assuming a more central role of the entire WM system, we could better understand the role of individual cognitive processes in relation to surgeon’s behavior and training outcome. Such an approach is deemed particularly useful if we wish to advance in the field of surgical education, as we know that learning surgery relies heavily on individual cognitive processes and domain-specific knowledge, and not merely on general knowledge and technical skills. Consequently, by supporting both technical and cognitive skill acquisition, we could potentially influence the rate and duration of learning, decreasing the learning curve for MIS. To put this idea into a practical context, one such cognitive training approach would be using a learning strategy called ‘chunking’. From a theoretical perspective, such a learning strategy involves breaking down a task(s) into multiple sequences/steps, by drawing on the individual own pre-existing knowledge and abilities. This is achievable through a ‘step-by-step’ process, by either breaking down large components of the task(s) or by focusing on breaking down a procedure in terms of a specific order and actions. Additionally, the same principle could be used to promote intraoperative learning, by, for example, teaching the resident how to ‘chunk’ visual information and teach them how to ‘fixate’ (through gaze training) on the only most important landmarks and cues on the screen. Such approach would in return teach the residents to appropriately allocate their attention resources, and thus in return increase the VWM capacity for information processing and storage, leading to more available capacity for decision-making, for example [34]. Yet another example of how to increase the capacity of VWM is through ‘cognitive rehearsal’, whereby the resident is asked to verbally describe each step of his action (say what they are thinking and doing), a strategy we called “think-aloud”. Through such method, the trainer would have a better understanding of the mental reasoning of the resident and could thus in return better understand the outcome behavior. We argue that such a holistic approach would be an effective research and training tool, as the residents would be taught to employ cognitive strategies that we know are used by expert surgeons [35]. Thus, considering that 97% of technical skill errors in resident surgeons are a result of disturbances in cognitive processing [36], one could argue that such a holistic approach could potentially accelerate the surgeons learning curve and promote competency-based training, all within the natural intra-operative environment.

## Conclusions

Spatial cognition was found to play an important role in MIS, with mental rotation showing significant influence over MIS learning and operative performance. Future research is not only encouraged to expand on this theoretical foundation but also to test its validity in practice. Acknowledging the role of the VWM in MIS potentially could considerably facilitate surgical education, as it would allow us to better understand how we ought to present visual information to the novice, using VWM aiding cues, in order to reduce cognitive load and increase skill acquisition costs. Finally, it should be noted that these conclusions are based on studies employing simulators. To which degree MR and VWM influence the surgeons’ abilities in the actual operating room remains to be determined. Future research is encouraged to further confirm and quantify these findings in the clinical settings. In the light of our findings, a closer exploration of how training the overall WM network could potentially improve and accelerate MIS learning should also be further explored.

## Abbreviations

MIS: Minimally invasive surgery
MR: Mental rotation
MRT: Mental rotation test
VSA: Visuo-spatial ability
VWM: Visual working memory
WM: Working memory
